# Health capabilities and the determinants of infant mortality in Brazil, 2004–2015: an innovative methodological framework

**DOI:** 10.1186/s12889-021-10903-9

**Published:** 2021-04-30

**Authors:** Alexandre Bugelli, Roxane Borgès Da Silva, Ladislau Dowbor, Claude Sicotte

**Affiliations:** 1 École de Santé Publique de l’Université de Montréal, student affiliated to the Centre de Recherche en Santé Publique (CReSP), 7101, Park Avenue, 3rd floor, Montreal (Québec) H3N, 1X9 Canada; 2 CAPES Foundation scholar (Coordenação de Aperfeiçoamento de Pessoal de Nível Superior Ministry of Education of Brazil, Science without Borders Program, proc. 12940/13-5), Brasilia, DF 700040-020 Brazil; 3 Ecole de Santé Publique de l’Université de Montréal (ESPUM), Centre de Recherche en Santé Publique (CReSP), 7101, Park Avenue, 3rd floor, Montreal (Québec) H3N 1X9, Canada; 4 Pontifícia Universidade Católica de São Paulo (PUC-SP), School of Economics and Business Administration Graduate Program, Rua Monte Alegre, 984, Perdizes, São Paulo, CEP 05014-901, Brazil; 5 École de Santé Publique de l’Université de Montréal (ESPUM), 7101, Park Avenue, 3rd floor, Montreal (Québec) H3N 1X9, Canada

**Keywords:** Infant mortality, Health capabilities, Public policies, Social determinants of health, Multilevel panel data with fixed effect nested within-clusters, Health economics

## Abstract

**Background:**

Despite the implementation of a set of social and health policies, Brazil has experienced a slowdown in the decline of infant mortality, regional disparities and persistent high death levels, raising questions about the determinants of infant mortality after the implementation of these policies. The objective of this article is to propose a methodological approach aiming at identifying the determinants of infant mortality in Brazil after the implementation of those policies.

**Method:**

A series of multilevel panel data with fixed effect nested within-clusters were conducted supported by the concept of health capabilities based on data from 26 Brazilian states between 2004 and 2015. The dependent variables were the neonatal, the infant and the under-five mortality rates. The independent variables were the employment rate, per capita income, *Bolsa Família* Program coverage, the fertility rate, educational attainment, the number of live births by prenatal visits, the number of health professionals per thousand inhabitants, and the access to water supply and sewage services. We also used different time lags of employment rate to identify the impact of employment on the infant mortality rates over time, and household income stratified by minimum wages to analyze their effects on these rates.

**Results:**

The results showed that in addition to variables associated with infant mortality in previous studies, such as *Bolsa Família* Program, per capita income and fertility rate, other factors affect child mortality. Educational attainment, quality of prenatal care and access to health professionals are also elements impacting infant deaths. The results also identified an association between employment rate and different infant mortality rates, with employment impacting neonatal mortality up to 3 years and that a family income below 2 minimum wages increases the odds of infant deaths.

**Conclusion:**

The results proved that the methodology proposed allowed the use of variables based on aggregated data that could hardly be used by other methodologies.

**Supplementary Information:**

The online version contains supplementary material available at 10.1186/s12889-021-10903-9.

## Background

### Context

Despite the implementation of a set of social and health policies aiming at improving the health of its populations [1], Brazil has experienced since 2009 a slower decline in infant mortality rates [2], recording major regional disparities and persistent high death levels [3, 4]. Such facts raise questions about the determinants of infant mortality after the implementation of those policies and their role as levers to grant a sustainable decline in infant mortality in Brazil.

*The* SUS *- Sistema Único de Saúde* (Brazilian Unified Health System) was created in 1988, with the implementation of the primary health program, the Family Health Strategy (FHS), in 1994. The FHS provided services delivered by multidisciplinary teams, comprising a physician, a nurse, a nurse assistant and community health professionals. A geographical area was assigned to each team which was responsible for the health of the population living in that area. All services were provided free of charge. In January 2017, the FHS counted on 39,709 teams, covering 97% (*n* = 5398) of the municipalities in Brazil [5].

In 2003, the Ministry of Social Development implemented the *Bolsa Família* Program (BFP) that provided monthly cash transfers to poor families in exchange for their complying with health and educational conditionalities. Those conditionalities required parents to ensure that children younger than 7 years of age to comply with a routine of growth monitoring and the childhood vaccination schedule and pregnant women and nursing mothers to attend prenatal care and nutrition education programs in a local healthcare provider. The educational conditionalities stipulated that children aged 6–17 were enrolled in school and maintained a minimum attendance rate according to their age bracket [6]. Since the implementation of the SUS and the FHS and BFP Programs, maternal and infant mortality declined [7], the fertility rate in poorer areas also decreased [8], life expectancy increased and a sharp decline in mortality due to transmissible diseases was recorded [9].

However, despite all those advancements, since 2009, the declining trend in infant mortality seems to have lost its momentum [2–4]. Between 2011 and 2016, Brazil experienced an economic and political crisis and in 2016 the declining trend of infant mortality was interrupted and increases in under-one and under-five-year-old mortality were observed in many regions of the country [2, 10, 11]. Those facts draw attention to possible effects of social determinants of health (SDH) that are beyond the reach of social and health policies and which may have influenced this change in the trend of infant mortality rates.

Maternal and child health are very closely related to social determinants of health that go beyond the impacts of adequate health services provision. Thus, infant mortality is also influenced by socioeconomic and living conditions factors such as income, employment and housing [12]. The infant mortality rate (IMR) is known as an indicator of population health, of health systems performance, and a useful tool for comparing social and health inequalities among populations [12–15].

In this sense, in addition to income and wealth growth that may improve living standards, Nussbaum places as fundamental capabilities being able to have bodily health, including reproductive health and making reproductive choices and to participate effectively in political choices [16]. According to the capabilities approach, inequalities in health result from gaps between the subjective freedom and substantive freedom of individuals. The notion of traditional economic development based on income and wealth does not capture the different dimensions of human development, which is, in fact, the means and the end of socioeconomic development. This point of view takes into consideration the real freedom of individuals as dependent on the expansion of their functional capacities, through access to essential resources such as the freedom of access to education, being in good health conditions, having access to healthcare, income and jobs, among other capabilities [16–19]. The concept of health capabilities is derived from the capabilities approach that was initially proposed by Nobel Prize in Economics Amartya Sen, but has been supported by numerous researchers that are dedicated to advancing knowledge about social justice and human development [16–22].

Many quantitative studies have been successful in establishing an association between the coverage rate of those health and social programs implemented in Brazil and different infant mortality indicators [6, 23–28]. However, those studies were dedicated exclusively to analyzing the combined effects of those programs between 1998 and 2010, when the country experienced a period of certain stability and economic growth, with rising employment and per capita income rates [29–31]. In addition, those studies relied on socioeconomic variables, such as per capita income, maternal schooling, and access to the safe water supply as control variables. Some of them were based on longitudinal and panel data analysis, with interpolated data for long periods, excluding a considerable number of municipalities in rural areas of the North macro-region of the country due to the unavailability of socioeconomic data until 2003 [28, 32], or limiting the analysis to a fraction of the totality of the municipalities of the country [25]. Also, none of those studies has attempted to use the capabilities approach to assess a possible association between SDH, such as employment or educational attainment, and infant mortality rates.

An important challenge to be addressed when using data in Brazil is the operationalization of variables based on data observed in different administrative instances over time. In Brazil, some data, especially employment and unemployment rates, are regularly estimated and disseminated only at the level of large metropolitan areas, states, and the country as a whole. Also, to use those data, one must consider the socioeconomic disparities among the macro-regions of the country. An additional challenge when using aggregated data is the risk of falling into the ecological fallacy and producing incorrect and biased estimations.

The objective of this article was to perform multilevel panel data with fixed effect nested within-cluster, based on the Conceptual Model of Health Capabilities CMHC as a methodological approach aiming at identifying the determinants of infant mortality in Brazil, after the implementation of FHS and BFP.

### The conceptual model of health capability (CMHC)

Under the perspective of Nussbaum, the concept of capabilities is closely related to rights that may be interpreted in a double sense. First, thinly and negatively, rights are preserved as long as the government does not interfere, or in a positive way such as by adopting the capabilities approach for which they require affirmative government support for creation and preservation [16].

According to the latter, the new Constitution enacted in Brazil in 1988 granted the right to health as a fundamental human right and an obligation of the State, launching the basis for the implementation of the SUS, conceived as a universal and equitable public health system aiming to provide health and social security to the entire population, and social and health policies such as BFP and FHS. The BFP was designed to interact with the FHS to increase both supply and demand for health services by motivating poor families to seek health and education through monetary incentives in exchange for complying with the program’s conditionalities [28].

**Fig. 1:**
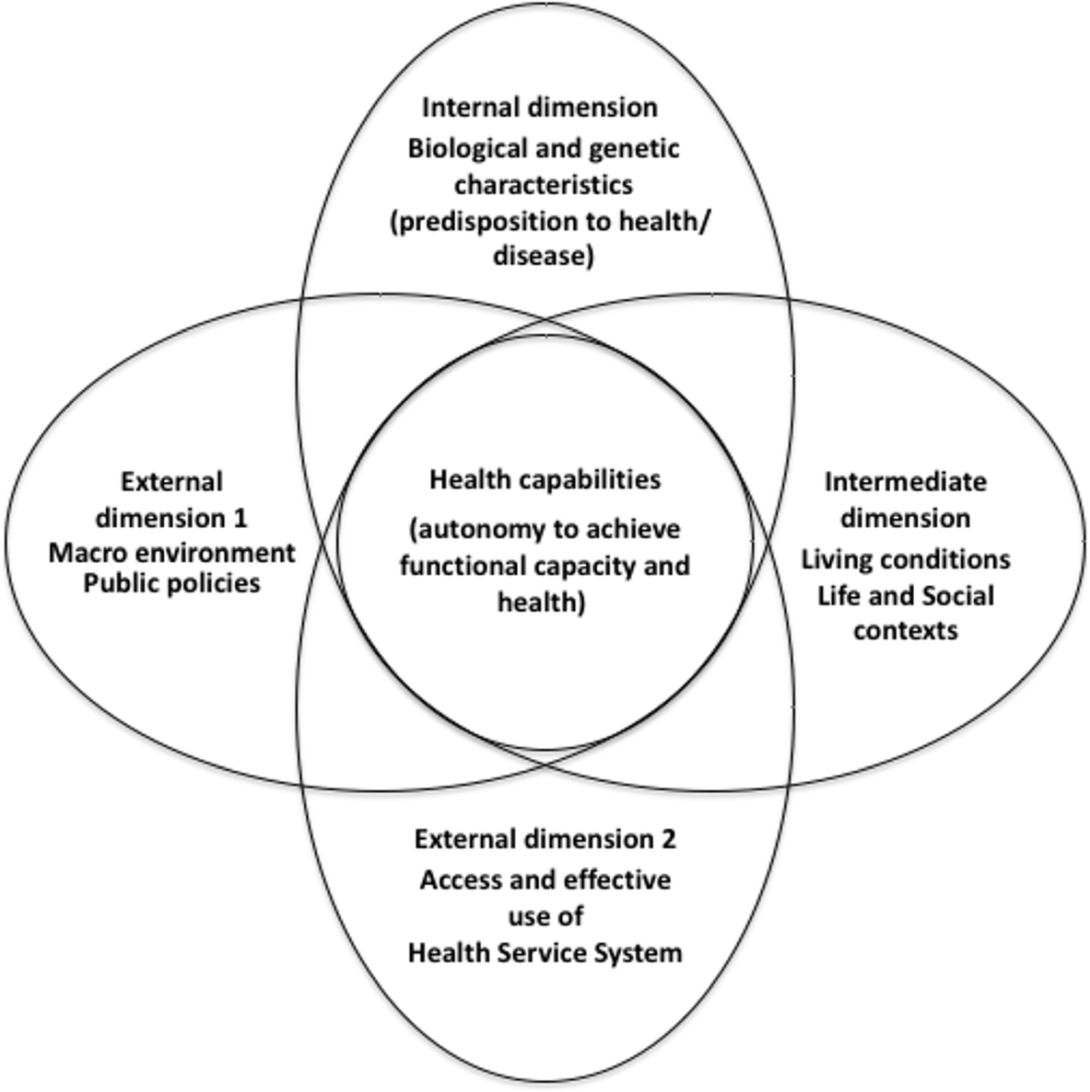
Conceptual Model of Health Capability (CMHC) adapted (Ruger, 2010)

In this regard, the CMHC (Fig. 1) has as its central idea that individuals seek both health and the ability to seek health [21]. Based on the concept of capabilities developed by Sen [17–19], the CMHC takes into account the individual’s sense of health and functional capacity for achieving health capability as the result of the interaction of three social dimensions, given one’s specific individual characteristics, the internal dimension.

The main assumption of the CMHC is that the individual’s health capabilities result from the interaction of four dimensions, one dimension referring to macro, social, political, and economic environment, the second related to the effective use of the health system, an intermediate dimension referring to the social and life contexts, given an internal dimension corresponding to the individual’s biologic and genetic predisposition to health/disease. In essence, the individual’s health capability is the ability to achieve health goals that an individual values, acting as his or her own health agent (health agency) and the health outcome itself as actions to keep and/or improve health (health functioning), both resulting from the interactions (arrangements) between the four dimensions of the CMHC. In this framework, there is a fine dividing line between state paternalism and self-agency as drivers to an individual for pursuing and keeping health as social and economic values.

The concept of health capabilities is increasingly becoming a valuable tool for the analysis of SDH. Furthermore, in this perspective, infant mortality has been identified as an adequate indicator of health attainment (health functioning), while the SDH such as education, housing, employment, and economic inequalities have been identified as social and environmental conversion factors (capabilities) [22].

Considering that the CMHC was developed as a tool for designing health policies and interventions in developed countries, some considerations must be made with respect to the specific characteristics of an emerging country when using this framework. Brazil is the largest Latin American country and the world’s fifth-largest, with a population of approximately 212 million people. Despite being considered an upper-middle-income country by the World Bank, the country ranks 73rd in terms of per capita income. Some social and structural aspects must be considered, such as high socioeconomic inequalities and low sewage services coverage rates (approximately only 60% of households on average are connected to the sewage collection and treatment network), for instance.

More as a disclaimer than an adaptation proposal, some aspects of our proposal to use the CMHC as an analysis tool should be highlighted. In the original model of the CMHC, external dimension 2 encompasses the influence of Public Health and health system and implicitly some aspects inherent to developed health systems, as financial equity and security. In this sense and considering the specificities of Brazil and that the basis of the capabilities concept is how people effectively live their lives, their freedom of choice (capabilities) and well-being (functionings), we consider income and socioeconomic inequalities, housing and overall living conditions as part of life circumstances in the intermediate dimension.

### Infant mortality, the Brazilian macro-regions, and data structure

Under the perspective of the health capabilities approach, to be able to have bodily health, reproductive health, making reproductive choices, and to control one’s social and physical environment, including to hold property and seek employment lie among the individual’s fundamental capabilities [16, 19, 20]. In this sense, infant mortality rates, our variables of interest, may be considered as indicators of population health attainment (health functioning) and education, employment, housing, access to health and economic inequalities as social and environmental conversion factors (capabilities).

**Fig. 2:**
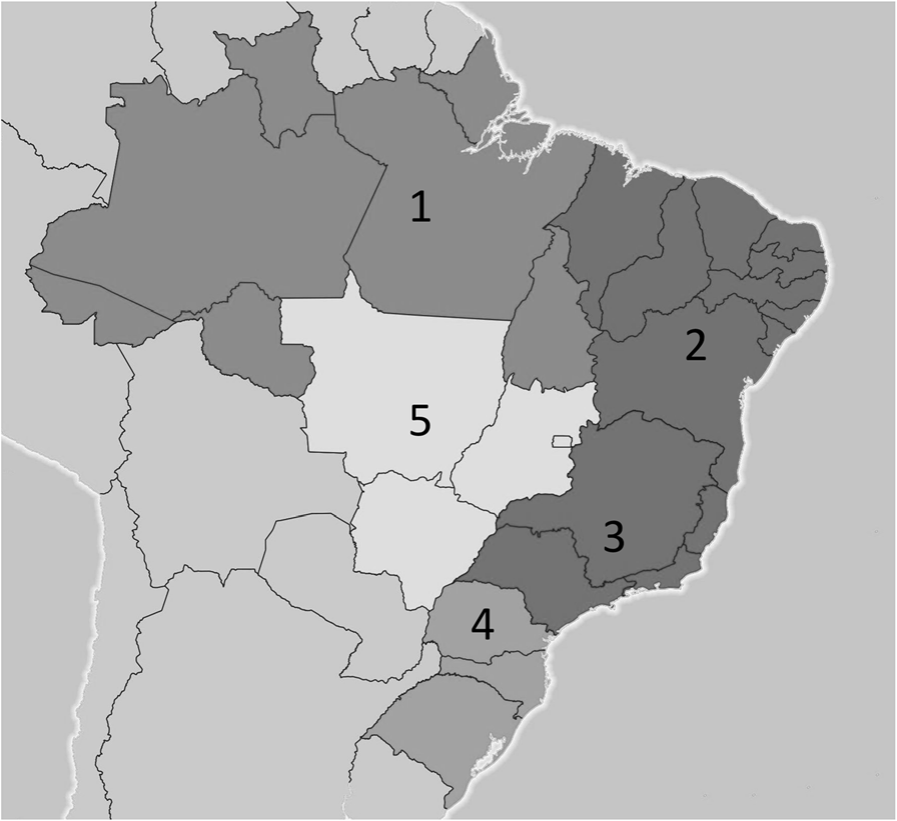
Brazilian macro-regions - (https://ibge.gov.br)

The 26 Brazilian states and the Federal District (FD) are grouped into five major macro-regions with very distinct socioeconomic, political, institutional, and cultural characteristics: North (1), Northeast (2), Southeast (3), South (4), and Midwest (5) (Fig. 2). Those socioeconomic characteristics are homogeneously distributed within macro-regions.

From the perspective of social determinants of health, identifying the factors affecting a health outcome requires the operationalization of variables that must fit for modeling multidimensional relations [33, 34]. To organize the possible factors acting on infant mortality among those different administrative instances over time, aiming to find statistically meaningful evidence when errors are differently distributed within and between macro-regions is a great challenge [33–35].

## Methods

### Panel and data specifications

#### Multilevel panel data

**Fig. 3:**
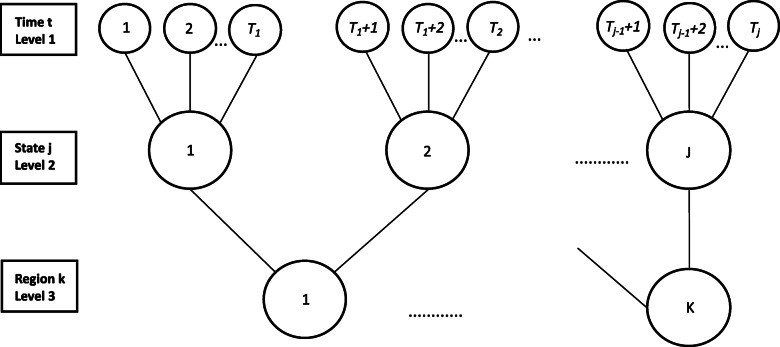
Three-level clustered structure with repeated measures. Adapted from: Hair & Favero, 2019 [36, 38]

According to Moulton, [36], modeling data from grouped structures based upon the assumption of independent disturbances is not appropriate. Individuals’ (units) observations over time within the aggregate level, as states nested in macro-regions, are clustered and are more similar to each other than units from another cluster. In this type of data structure there are clustered errors and they occur because unobserved factors varying over time are more homogeneous among clustered units than others and there are different levels of fixed effects within and between clusters [37]. The statistical study with such data structure (Fig. 3) demands multilevel clustering panel data [38].

In this data structure, the errors are not i.i.d. and the within-cluster errors (υ_tjk_) may occur and are broken down in a common choke component in a given observation (ν_k_, cluster-specific error) and an idiosyncratic component (ζ_jk_), as follows:

$$ {\upupsilon}_{\mathrm{k}}={\upnu}_{\mathrm{k}}+{\upzeta}_{\mathrm{jk}} $$

#### VCE (variance-covariance matrix of estimators)

The absence of clustering control may lead to underestimated standard errors and overestimated statistical significance. In this sense, by relaxing the assumption of i.i.d. errors, opting for a more realistic data structure that allows intragroup correlations, we should consider the use of a cluster-robust variance-covariance matrix of estimators (VCE) [39, 40].

#### Data structure, variables, and data sources

We created a secondary aggregated database from 12 periods (years) between 2004 and 2015 and having as units of analysis the 26 Brazilian states, distributed among the 5 socioeconomic macro-regions (Fig. 2). The country’s capital, Brasília, is a hybrid administrative instance (city-state) which presents a disproportionate per capita income when compared to other states, which may introduce bias in our models. Therefore, Brasília was excluded from our study. The study period was defined considering the beginning of BFP that was officially implemented in October 2003, as well as the availability of data regarding the employment rate. We used the data series collected and disseminated by the PNAD (National Household Sample Survey). In 2012, the PNAD evolved to PNAD Continuous, on a monthly basis, and in 2015, the Brazilian Institute of Geography and Statistics (IBGE) stopped publishing the former PNAD annual series referring to employment rates. By coding the states (id: from 1 to 26) and macro-regions (mr: from 1 to 5), we attributed categorial variables in the model to nest states (id) within-clusters (mr and years). We relied on 312 observations from 26 Brazilian states over 12 years, nested in 60 clusters (5 macro-regions times 12 years). Our dataset is balanced, implying that observations are corresponding to all units over our study period (T2 – T1 = … TJ – TJ-1).

#### Dependent variables

Our dependent variables were the neonatal mortality rate (NMR), infant mortality rate between 0 and 1 year (IMR), and infant mortality rate between 0 and 5 years (U5MR). Those indicators are widely used in infant mortality studies. This will allow us to compare our results with those of other studies. The dependent variables were indicators of the population’s health attainment (health functioning).

#### Independent variables

According to a scoping review based on the CMHC, we identified possible determinants of infant mortality acting simultaneously with the social and health policies implemented in the country since 2004. The capabilities approach suggests that the employment/unemployment rate is one of the conversion factors (capabilities) of health functioning (infant mortality) among the factors accounting for the infant mortality rate. We decided to use the employment rate (occupancy rate) rather than the unemployment rate due to methodological changes in the estimation and publicity of the unemployment rate in Brazil in recent decades. For surveys carried out between 1983 and 2002, IBGE considered the population at working-age (PWA) to be those over fifteen years of age. According to the IBGE’s new methodology, over 10 years old were part of the working-age population. In defining the employed or unemployed population, IBGE considered the minimum limit of 15 h per week for unpaid work, while the new survey included those who worked at least 1 h a week.^1^ Also, the capabilities approach is a positively conceived concept in the sense that one of the basic capabilities is a person’s freedom to be able to seek employment. In addition to using the annual employment rate (OCC), we used three different time lags. The variables OCC, OCC1, OCC2 and OCC3 referred to the employment rate by considering zero-, one-, two- and three-years-time lags respectively, in relation to both the dependent and other independent variables. This strategy aimed at determining to which extent the employment rate may impact infant mortality. In an econometric study on the economic fluctuations in the USA as well as infant and maternal mortality, Brenner [41] demonstrated that in industrialized countries, the association between the unemployment rate and infant mortality can vary between 0 and 5 years and that the optimal time lag lies between 1 and 2 years, depending on the infant mortality. Per capita income (represented by the Real Gross Domestic Product per capita) is also considered an important conversion factor related to both the macro-environment (as the result of macroeconomic management and policy) and the intermediate dimension (socioeconomic inequalities and living conditions). We also stratified income by number the average nominal minimum wages earned by households as an attempt to identify income inequalities that may be affecting infant mortality indicators. This variable was stratified according to the following categories: low-income: from 0 to 1 minimum wage (IS_F < 1 MW); low-medium income: 1 to 2 minimum wages (1 MW < IS_E < 2 MW); medium-income: from 2 to 5 minimum wages (2 MW < IS_D < 5 MW); medium-high income: from 5 to 10 minimum wages (5 MW < IS_C < 10 MW); high-income: 10 to 20 minimum wages (10 MW < IS_B < 20 MW) and very high income: more than 20 minimum wages (IS_A > 20 SM). The coverage rate of the PBF was used to assess the effect of a macro-environment-related social policy on the different infant mortality indicators. The fertility rate was a control variable. The variables concerning safe water supply and sewage services were used to evaluate housing and living conditions as factors of the intermediate dimension impacting infant mortality rates. The number of live births by prenatal visits was a proxy of the effectiveness and quality of prenatal care. We also conducted estimations using the number of physicians and nurses by thousand inhabitants, which was designed in order to assess the impact of the availability of health professionals on infant mortality. This variable may also be interpreted as a proxy to evaluate the access and comprehensiveness of healthcare, regardless of the availability of health facilities. The educational attainment ratio is an important variable in our conceptual framework. Like the dependent variables that measures infant mortality as a health functioning variable, educational attainment measures educational functioning. Both infant mortality and educational attainment are capabilities “converted” into functionings. Although in the original CMHC there are no references of educational attainment, it is reasonable to suppose that there is a connection between the development of health and educational capabilities at the household level as a result of the interaction of the four social dimensions of the CMHC.

For the variables already expressed in unit values, such as the number of live births by prenatal visits, the number of nurses per thousand inhabitants, the fertility rate, as well as dependent variables NMR, IMR and U5MR, no transformations were applied. For all variables expressed in percentages, such as employment rate (OCC), the proportion of family income expressed in minimum wages (IS_A to IS_F), the coverage rate of the BFP, sewage services and water supply coverage rates, we divided the percentage values by 100 to convert them also into unit values. Regarding household per capita income (RGDP), this figure was divided by one thousand. As those are linear transformations, they do not affect estimations, but allow a better analysis of the results in terms of magnitude.

For the Census Year of 2010, PNAD surveys were not conducted and there were no values for some variables in that specific year since the IBGE uses different samples and methodology for Census and PNAD. Thus, for employment (OCC), per capita income (RGDP), household income stratified by the number of average nominal minimum wages (IS_A to IS_F), water and sanitation data, total safe water coverage (WCT), sewage collection and treatment coverage (SWT), and educational attainment (EDA) we applied linear interpolation to obtain the values for 2010.

The dependent and independent variables and their definitions are displayed in Table 1. according to the dimensions of the CMHC.

**Table 1:** Variables according to the CMHC

Variable name	Abbreviation	Description/ expected signal	Data source	Dimension according to the CMHC
**Neonatal mortality rate**	**NMR**	The ratio of the children who died during the first 28 days of life and those born alive in a given year	Ministry of Health/DATASUS	Dependent
**Infant mortality rate**	**IMR**	The ratio of children who died under-one-year of age to those born alive in a given year	Ministry of Health/DATASUS	Dependent
**Under-five mortality rate**	**U5MR**	The ratio of children who died under-five-years of age to those born alive in a given year	Ministry of Health/DATASUS	Dependent
**Employment rate**	**OCC**	The ratio between the total of employed population aged 10 years or more (occupation rate) and the total of the economically active population	IBGE/PNAD Survey	External 1
**Income per capita (Real GDP)**	**RGDP**	Deflated Gross Domestic Product (RGDP) of a state divided by its number of inhabitants in a given year	IBGE/PNAD Survey	External 1
**Household income according to minimum wage strata**	**IS_A to IS_F**	The ratio between the income measured in terms of the minimum wage earned by households and the total households in the state	IBGE/PNAD Survey	Intermediate
***Bolsa Família*** **program**	**BFP**	The proportion between the families followed up by the BFP and the number of families to follow in a given year	Ministry of Health/DATASUS	External 1
**Fertility rate**	**FR**	The ratio between live births in a given year and the total female population at reproductive age (between 15 and 49 years)	Ministry of Health/DATASUS	Intermediate
**Safe water supply**	**WCT**	The proportion of total households with access to safe water supply service in relation to the total households in the state in a given year	IBGE/PNAD Survey	Intermediate
**Sewage services**	**SWT**	The proportion of total households with access to sewage services in relation to the total households in the state in a given year	IBGE/PNAD Survey	Intermediate
**Quality of prenatal care**	**LBPRE**	The proportion of live births by the state in a given year by the number of prenatal visits of women at reproductive age in the state in a given year	Ministry of Health/DATASUS	External 2
**Access to health professionals**	**MEDEN**	The sum of the average number of physicians plus the average number of nurses in a given year divided by thousand inhabitants living in a state	Ministry of Health/DATASUS	External 2

#### Data sources

All infant mortality rates were obtained by computing data directly from infant deaths recorded in the SIM and births recorded in SINASC systems provided by DATASUS database (TABNET) without any further adjustment. Even though certain references highlight the limitations for the direct calculation of infant mortality rates such as underreporting [42, 43], we adopted this approach supported by the fact that an important question regarding indirect method to calculate infant mortality rate is that corrections are made building on data from relatively small samples and/or census surveys which tends to smooth the trend of infant mortality over time and attenuate short term variations, which is a very undesirable factor to longitudinal analysis. Estimates derived from forecasts relied on the adjustments applied to observed historical data do not take into account the effect of short-term changes resulting from health and social programs and may hide their influence on infant mortality. Further, they may also hide the real impact of economic and political crisis on infant mortality rates. Many longitudinal and ecological studies have been done also based directly on data from the Ministry of Health’s mortality information systems (SIM) and live births (SINASC) [1, 6, 26, 44–47].

The employment rate (OCC), the fertility rate (FR), the school attainment rate (EDA), Real Gross Domestic Product per capita (RGDP), and the household income stratified by socioeconomic categories (IS) were obtained from the database of IBGE.^2^ Those data were estimated through the PNAD survey. The PNAD was conducted annually by the IBGE since 1981 and surveyed several characteristics of the population such as household structure, education, labor, income, and fertility. The PNAD sample in 2012 consisted of 147,203 households, with 362,451 residents.

For the Census Year of 2010, PNAD surveys were not conducted and there were no values for some variables in that specific year since the IBGE uses different samples and methodology for Census and PNAD. Thus, for employment (OCC), per capita income (RGDP), household income stratified by the number of average nominal minimum wages (IS_A to IS_F), water and sanitation data, total safe water coverage (WCT), sewage collection and treatment coverage (SWT), and educational attainment (EDA) we applied linear interpolation to obtain the values for 2010).

Data on dependent variables (NMR, IMR and U5MR), as well as data on the families followed by the BFP and the number of live births in relation to the number of prenatal visits, and the proportion of physicians and nurses per thousand inhabitants, were obtained from the database of the Brazilian Ministry of Health, DATASUS.^3^

For the year 2004, there were no data available in the DATASUS for the number of families covered by the BFP and for the number of physicians and nurses (MEDEN). We used backward linear regression forecasting (“backcasting” in fact) to generate values for the number of physicians and nurses for that year. For BFP coverage specifically, as the program was implemented in October 2003, we applied data only from the period when the program had expanded from 2005 to 2009 to estimate values for 2004.

#### Statistic model

**Fig. 4:**
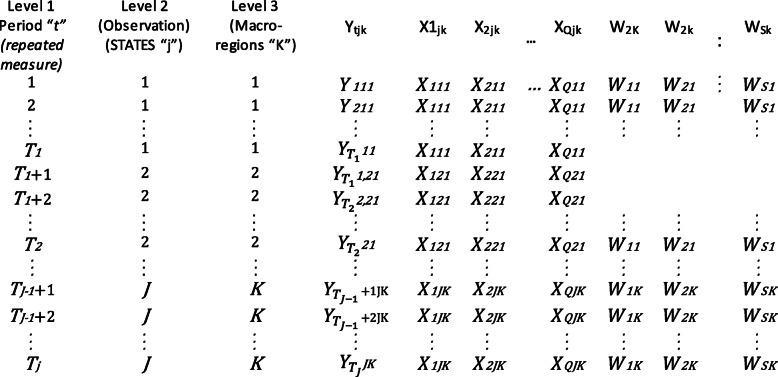
Panel data with three level structure notation. Adapted from: Hair & Favero, 2019 [36, 38]

The general panel data model for our three-level dataset structure is noted in Fig. 4 as follows:

The general statistical model specification observes the following formulation:

*Y*_*ijt*_ *= α*_*ijt*_ *+ β*_*1*_*OCC*_*ijt*_ *+ β*_*2*_*RGDP*_*ijt*_ *+ β*_*3*_*BFP*_*ijt*_ *+ β*_*4*_*FR*_*ijt*_ *+ β*_*5*_*EDA*_*ijt*_ *+ β*_*6*_*LBPRE*_*ijt*_ *+ β*_*7*_*WCT*_*ijt*_ *+ β*_*8*_*SWT*_*ijt*_ *… + υ*_*ij*_ *+ ε*_*ijt*_, where: “Yijt” is the result of the unit (scalar) at time “t”, “αijt” is the intercept specific to each unit and: “OCCijt”; “RGDPijt” (or alternatively: “ IS_Aijt “,“ IS_Bijt “,“ IS_C ijt “,“ IS_Dijt “,“ IS_Eijt “,” IS_Fijt”); “BFPCOVijt”; “FRijt”, “EDAijt”; “LBPREijt”; “WCTijt” and SWTijt” are the vectors (1 x K) of the covariant which vary over time. The β’s are the vectors of the coefficients and “υ_ij_ “_=_ ν_k_ + ζ_jk_, is the decomposed scalar of the clustered fixed effects and “ε_**ijt**_” is the error term.

This model considers the parameters and specifications established by the “reghdfe” command of statistical software Stata©, version 13, for multilevel panel data.

### Statistical analysis

First, we calculated a correlation matrix (Appendix 1) with all variables to evaluate the correlation between the independent and dependent variables and the signals of those correlations to compare those results with the estimations.

As discussed previously, our dataset structure is based on different levels of fixed effects. We opted for estimating multilevel linear regressions panel data using Correia’s [48] multilevel panel data with fixed effect nested within the cluster model. By using Correia’s multilevel panel data with fixed effect nested within the cluster model we can treat observations at different levels of homogeneity. Further, by absorbing categorical variables, as time (years), and clustering combined fixed effects levels as time and macro-regions (mr), as “years” and “mr”, we did not double penalize the robust standard errors when computing the absorbed degrees of freedom. Appendix 2 shows the general model for infant mortality rate (IMR) and employment rate with one-year-time lag (OCC1), absorbing year.

### Study design

We conducted 4 sets of estimations with 33 data panels with fixed effect nested within-cluster models alternating between the three dependent variables (NMR, IMR, and U5MR) and their possible association with the independent variables. In the first set, 12 panels combined the three alternatives of time lags for employment rate (OCC, OCC1, OCC2, and OCC3) to identify if the three dependent variables (NMR, IMR, and U5MR) were associated with employment rate, and, if so, what was the optimal time lag according to the specific infant mortality indicator. After identifying the best adjusted time lag for a possible association between employment and all infant mortality rates we estimated a general model using all other covariates: per capita income (RGDP), BFP coverage (BFP), the fertility rate (FR), educational attainment (EDA), total live births by the number of prenatal visits (LBPRE), water supply coverage (WST) and sewage services coverage (SWT). For the income stratified (from ISA to ISE), in the third set, we ran 18 panels combining the three dependent variables (NMR, IMR, and U5MR). Finally, we conducted 3-panel models in the fourth set to compare the effect of the availability of physicians and nurses (MEDEN) and the variable live births by the number of prenatal visits (LBPRE) in the general model to assess the effective use and access to the health system in association with the dependent variable (NMR, IMR and U5MR).

## Results

After estimating the 33 models, we present the main results and comments in the tables below. Table 2 summarizes the results of the first set of estimations using the different time lags for employment rate (OCC, OCC1, OCC2, and OCC3) and our dependent variables: neonatal mortality rate, infant mortality rate, and under-5 mortality rates. We controlled all models for other covariates: Real GDP per capita, BFP coverage, fertility rate, educational attainment, number of live births by prenatal visits and safe water supply and sewage services coverage rates.

**Table 2:** Estimations using different time lags for employment rate (OCC)

	Neonatal mortality	Infant mortality	Under-five mortality
	Coef. (95% CI) ***R***^**2**^ ***p***-value	Coef. (95% CI) ***R***^**2**^ ***p***-value	Coef. (95% CI) ***R***^**2**^ ***p***-value
**Employment without time lag**	−17.50 (− 29.53, − 5.47) 0.60 0.005	−17.27 (− 29.97, − 4.56) 0.74 0.009	−10.94 (− 30.93, 9.05) 0.71 0.278
**Employment with 1-year time lag**	−17.73 (− 28.98, − 6.49) 0.60 0.003	−19.18 (− 30.94, − 7.43) 0.74 0.002	−17.63 (− 31.32, − 3.95) 0.71 0.012
**Employment with 2-years time lag**	−19.95 (− 32.23, − 7.68) 0.61 0.002	−12.83 (− 23.66, − 2.00) 0.73 0.021	−16.11 (− 27.99, − 4.24) 0.71 0.009
**Employment with 3-years time lag**	−20.57 (− 32.05, − 9.08) 0.61 0.001	−11.48 (− 21.99, − 0.96) 0.73 0.033	−9.87 (− 22.76, 3.02) 0.71 0.131

*R*^2^ refers to adjusted values

The estimations with different infant mortality rates show that the employment rate is best adjusted to IMR with a one-year time lag (*p*-value: 0.002, CI: − 30.94; − 7.43 and adj. *R*^2^: 0.74) and with a two-year time lag for under-five mortality (*p*-value: 0.009, CI: − 27.99; − 4.24 and adj. *R*^2^: 0.71). Curiously, for the association between the employment rate and NMR, the estimations show that there is a gradient from the less statistically significant association, with no time lag (*p*-value: 0.005, CI: − 29.53; − 5.47 and adj. *R*^2^: 0.60) to the most statistically significant, with three-year-time lag (*p*-value: 0.001, CI: − 32.05; − 9.08 and adj. *R*^2^: 0.61).

Table 3 presents the estimations for the general model with all other covariates and their association with infant mortality rates. The estimations were made using a general panel data model with a one-year time lag for employment rate since it was the time range that most fitted the models, the Real GDP per household, the BFP coverage, fertility rate, educational attainment ratio, number of live births by prenatal visits and safe water supply and sewage services coverage rate.

**Table 3:** General model with one-year employment rate time lag

	Neonatal mortality	Infant mortality	Under-five mortality
	Coef. (95% CI) ***R***^**2**^ ***p***-value	Coef. (95% CI) ***R***^**2**^ ***p***-value	Coef. (95% CI) ***R***^**2**^ ***p***-value
**Employment with one-year time lag**	− 17.73 (− 28.98, − 6.49) 0.60 0.003	− 19.18 (− 30.94, − 7.43) 0.74 0.002	−17.63 (− 31.32, − 3.95) 0.71 0.012
**Per capita income** **(RGDP)**	− 0.11 (− 0.15, − 0.07) 0.60 0.000	− 0.07 (− 0.12, − 0.01) 0.74 0.017	− 0.08 (− 0.15, − 0.17) 0.71 0.015
***Bolsa Família*** **coverage rate**	− 5.88 (− 9.18, − 2.59) 0.60 0.001	− 4.83 (− 7.43, − 2.23) 0.74 0.000	− 3.65 (− 6.72, − 0.60) 0.71 0.020
**Fertility rate**	0. 67 (− 0.02, 1.38) 0.60 0.056	3.19 (2.25, 4.12) 0.74 0.000	4.00 (3.00, 5.00) 0.71 0.000
**Educational attainment**	−3.85 (−6.20, − 1.50) 0.60 0.002	− 7.13 (− 10.20, − 4.07) 0.74 0.000	− 8.23 (− 11.59, − 4.87) 0.71 0.000
**Live-births by prenatal visits**	− 1.34 (− 2.58, − 0.11) 0.60 0.034	− 1.33 (− 2.81, 0.15) 0.74 0.076	− 0.93 (− 2.48, 0.62) 0.71 0.233
**Safe water supply coverage rate**	0.76 (− 0.08, 1.61) 0.60 0.077	0.54 (− 0.45, 1.53) 0.74 0.279	− 0.59 (− 1.79, 0.60) 0.71 0.326
**Sewage servive coverage rate**	− 0.32 (− 0.91, 0.28) 0.60 0.291	0.32 (− 0.44, 1.07) 0.74 0.407	− 0.65 (− 1.55, 0.25) 0.71 0.154

*R*^2^ refers to adjusted values

As already mentioned, there is an important effect of employment on almost all infant mortality indicators, especially on IMR. Per capita income also has a statistically significant association with all infant mortality indicators and is better adjusted to NMR (*p*-value: 0.000, CI: − 0.15; − 0.07 and adj. *R*^2^: 0.60). The association between the BFP and infant mortality rates is also important and is better adjusted to NMR (*p*-value: 0.001, CI: − 9.18; − 2.59 and adj. *R*^2^: 0.60) and IMR (*p*-value: 0.000, CI: − 7.43; − 2.23 and adj. *R*^2^: 0.74) than to U5MR (*p*-value: 0.020, CI: − 6.72; − 0.60 and adj. *R*^2^: 0.71). Fertility is positively associated with IMR (*p*-value: 0.000, CI: 2.25; 4.12 and adj. *R*^2^: 0.74) and U5MR (*p*-value: 0.000, CI: 3.00; 5.00 and adj. *R*^2^: 0.71). Educational attainment is strongly associated with all infant mortality indicators with greater statistical significance for IMR and U5MR (NMR: *p*-value: 0.002, CI: − 6.20; − 1.50 and *R*^2^: 0.60, IMR: *p*-value: 0.000, CI: − 10.20; − 4.07 and adj. *R*^2^: 0.74 and U5MR: *p*-value: 0.000, CI: − 11.59; − 4.87 and *R*^2^: 0.71). Live births by prenatal visits are only statistically significant for NMR (*p*-value: 0.000 *p*-value: 0.034, CI: − 2.58; − 0.11 and adj. *R*2: 0.60). The safe water supply and sewage services coverage rates are not associated with any infant mortality indicator in the general model.

Table 4 presents the results of the estimations using household stratified by minimum wage, controlled by the other independent variables. An association between household income and NMR is confirmed for almost all income strata. This association is less significant for the highest household income stratum. There is no association between the highest stratum, above 20 minimum wages, approximately US$ 5300.00 at 2015 US$ current prices, with IMR and U5MR rates (*p*-value: 0.135, CI: − 64.58; 8.92 and adj. *R*^2^: 0.73, and *p*-value: 0.594, CI: − 55.12; 31.82 and adj. *R*^2^: 0.70, respectively). It is worth noting that there is a signal inversion depending on the specific income strata and infant mortality rate. The signal is negative for the proportion of households below two minimum wages, suggesting that an income under this income bracket presents an increased risk for all infant mortality. On the other hand, an income over two minimum wages would function as a protective factor for infant mortality, except for IMR and U5MR in the highest-income strata. It is worth noting that for a household income between 2 and 5 minimum wages, the association between income and U5MR is weakly significant (*p*-value: 0.051, CI: − 21.66; 0.03 and adj. *R*^2^: 0.71).

**Table 4:** The relation between child mortality and household income stratified by minimum wage

	Neonatal mortality	Infant mortality	Under-five mortality
	Coef. (95% CI) ***R***^**2**^ ***p***-value	Coef. (95% CI) ***R***^**2**^ ***p***-value	Coef. (95% CI) ***R***^**2**^ ***p***-value
**% of households living with an income above 20 minimum wages**	−53.44 (− 90.09, −16 .77) 0.57 0.005	−27.83 (− 64.58, 8.92) 0.73 0.135	− 11.64 (− 55.12, 31.82) 0.70 0.594
**% of households living with an income between 10 and 20 minimum wages**	−27.60 (− 42.86, − 12.35) 0.58 0.001	−25.27 (− 43.46, − 7.08) 0.74 0.007	−34.40 (− 66.86, − 1.94) 0.71 0.038
**% of households living with an income between 5 and 10 minimum wages**	−11.87 (− 17.86, − 5.87) 0.58 0.000	−13.31 (− 21.26, − 5.36) 0.74 0.001	−17.25 (− 29.67, − 4.83) 0.72 0.007
**% of households living with an income between 2 and 5 minimum wages**	−6.94 (− 11.99, − 1.89) 0.57 0.008	−11.17 (− 18.11, − 4.25) 0.74 0.002	−10.82 (− 21.66, 0.03) 0.71 0.051
**% of households living with an income between 1 and 2 minimum wages**	10.70 (5.00, 16.40) 0.58 0.000	9.46 (1.19, 17.74) 0.74 0.026	13.44 (3.05, 23.82) 0.71 0.012
**% of households living with an income between 0 and 1 minimum wage**	7.53 (3.90, 11.16) 0.58 0.000	9.16 (4.06, 14 .27) 0.75 0.001	8.87 (1.03, 16.71) 0.71 0.027

*R*^2^ refers to adjusted values

The relation between the employment rates and infant mortality can be interpreted as a confounding factor due to the relation between income and infant mortality rates. However, this possibility should be observed with caution, mainly because a considerable proportion of income refers to types of income other than wages, such as social programs and retirement benefits, profits, interests, dividends, rents, and royalties. Also, estimations demonstrated that the signal of the relation between income and mortality rates may change, depending on the household income strata. It is hardly possible to suppose that the employment rate stratified by worked hours follows the same signal inversion in relation to infant mortality rates. Those findings suggest that employment and income have different impacts on child mortality.

We also conducted estimations using the total live births per year according to the number of prenatal visits in the population of women aged 15 to 49 and the proportion of physicians and nurses per thousand inhabitants (Table 5).

**Table 5:** The relation between the proportion of live births by prenatal visits and the proportion of physicians and nurses per thousand inhabitants and child mortality

	Neonatal mortality	Infant mortality	Under-five mortality
	Coef. (95% CI) ***R***^**2**^ ***p***-value	Coef. (95% CI) ***R***^**2**^ ***p***-value	Coef. (95% CI) ***R***^**2**^ ***p***-value
**Live-births by prenatal visits**	−1.34 (− 2.58, − 0.11) 0.60 0.034	−1.33 (− 2.81, 0.15) 0.74 0.076	−0.93 (− 2.48, 0.62) 0.71 0.233
**Number of physicians and nurses by 1000 inhabitants**	− 0.42 (− 0.81, − 0.02) 0.60 0.041	−0.93 (− 1.32, − 0.54) 0.75 0.000	−0.87 (− 1.26, − 0.48) 0.72 0.000

*R*^2^ refers to adjusted values

The number of live births is statistically significant for NMR only (*p*-value: 0.034, CI: − 2.58; − 0.11 and adj. *R*^2^: 0.60). This association has a negative signal, denoting that the lower the number of prenatal visits in relation to the absolute number of live births the higher the efficiency of prenatal care. On the other hand, the availability of physicians and nurses is statistically significant for all infant mortality indicators, with a negative signal as well (*p*-value: 0.042 *p*-value: 0.041, CI: − 0.81; − 0.02 and adj. *R*^2^: 0.60 for NMR, *p*-value: 0.000, CI: − 1.32; − 0.54 and adj. *R*^2^: 0.75 for IMR and *p*-value: 0.000, CI: − 1.26; − 0 .48 and adj. *R*^2^: 0.72 for U5MR), denoting that the access to health professionals has an impact on all child mortality indicators, especially IMR and U5MR.

The 33 models indicated that the multilevel panel data with fixed effects nested within-cluster based on observations grouped in regions with very different socioeconomic characteristics proved to be an efficient method for identifying and analyzing the determinants of infant mortality in Brazil.

The results show that in addition to the variables already known in previous studies to be associated with infant deaths, such as coverage of the BFP, per capita income and fertility rate [6, 24–26], other factors may have important effects on child mortality. The employment rate, educational attainment, quality of prenatal care and population access to health professionals are also elements impacting infant health from birth to 5 years old.

The results also suggest that a family income slightly higher than 2 minimum wages can make a difference in avoiding infant deaths. In contrast, it is also worth mentioning that those findings highlight the importance of the BFP to prevent child mortality, considering that a minimum financial improvement that can have a great impact as a protective factor for families living on much less than 1 minimum wage. The safe water supply and sewage services coverage rates were not associated with any infant mortality indicator. Those results will be discussed in the next section.

## Discussion

The results of this study proved that the conceptual framework adopted, the CMHC, is a useful tool for the analysis of the effects of social determinants of health in an upper-middle-income country, but with distinct subregional characteristics, under the effects of an inclusive institutional, social and health policies framework. Those results were only possible with the use of the multilevel panel data model with fixed effects nested within-cluster. The method presented allowed the use of the variables provided by the conceptual framework by applying aggregated data that could hardly be used by other methodologies without leading to incorrect estimations. Our models were able to isolate the effects of the variables under study from factors not observed, which are subject to estimation errors due to different degrees of error homogeneity within and between clusters.

Although other studies on infant mortality in Brazil relied on larger datasets for the analysis of the factors impacting infant mortality in Brazil after the implementation of FHS and BFP, our models relied on a longer observational window that allowed us to infer more about specific factors related to infant mortality rates such as the relation between the employment rate and different indicators of infant mortality and between the BFP and the neonatal mortality rate or the threshold of household income according to minimum wage bracket which acts as a protective factor for infant mortality. Furthermore, the use of the methodology of clustered observations at different levels of fixed effects is a low cost-benefit solution, since it relies on a small volume of data when compared to conventional panel data studies.

In this section, we will address each of the variables and their relationship with mortality rates and conduct the analysis in line with CMHC.

### Employment and infant mortality

Regarding the health capabilities approach, the findings may be interpreted as a possible effect of employment on the childbearing decision as part of reproductive choice at the household level, which may be a result of a reasonable period of increasing employment that impacted a substantial number of households and therefore neonatal mortality that represents more than 70% of all infant mortality rates. On the other hand, the association between a one-year time lag of employment rate and IMR that represents almost 90% of the total U5MR [49], may be related to a better socioeconomic condition and the household ability, or freedom, to child-caring, feeding, identifying an emergent health issue and searching for best treatments for death prevention.

Few studies have addressed the relation between employment and unemployment and infant mortality in Brazil. In a study using a panel data over populational health and economic downturn in Latin America, Williams et al. [50] found that besides income and inflation, unemployment is also strongly related to under-5 mortality. Although the authors reported that unemployment data in Brazil were not available for the study period (1981 to 2010). In a mixed study with data based on interviews collected in a small town near São Paulo, Ventura et al. [51] concluded that among adults who lived in the same household, the fact of having or not having a job was an important factor in determining the degree of stability and vulnerability of families, which is not in disagreement with the capabilities approach.

### Income and infant mortality

Changes in the income signals according to strata and different effects on infant mortality may be related to the association between income and access to health services. The change of signal above two minimum wages stratum suggests that the higher the proportion of families earning up to 2 minimum wages on average (about US$ 525.00 in 2015 at current prices), the higher infant mortality tends to be, except for infant mortality (IMR) and under-five mortality (U5MR) and stratum “A” household income, that are not significantly associated. Therefore, a household income of less than two minimum wages increases the odds of infant death and a slight improvement in household income over 2 minimum wages may have a considerable impact on infant mortality in all age brackets.

Such results suggest that an income threshold above two minimum wages per household provides more freedom to prevent infant deaths.

In a geospatial study on the inequality of infant mortality in Brazil, conducted between 2006 and 2010, Oliveira et al. [26] concluded that low household income, fewer prenatal visits and fewer neonatal intensive care unit beds are correlated, forming a cluster in the North and Northeast macro-regions of the country.

### *Bolsa Família* program and infant mortality

Our findings are in accordance with the results of other studies. Many authors have highlighted the importance of the BFP in reducing socioeconomic inequalities that hinder the access to primary healthcare provided by the FHS and improving nutritional status with positive effects on infant health and mortality [5, 25, 28].

Nevertheless, some studies show that the interaction between the BFP and the Family Health Program (FHP) is associated with higher average prenatal visits only in the Northeast states [28] and that the BFP has little or no impact on neonatal mortality [6]. Other studies confirmed the impacts of BFP on IMR and U5MR [5, 25]. Those findings are somewhat controversial, considering that another study pointed that increased neonatal mortality in the Northeast macro-region between 2006 and 2010 was linked to lower numbers of prenatal visits and socioeconomic conditions [26].

An aspect to be considered when analyzing those differences is that our study was conducted over a 12-year period after the implementation of the BFP and that those studies were conducted over shorter periods, from 5 to 7 years after the implementation of the BFP [6, 25] or in ecological analysis with predefined periods or over a single period [5, 25]. Our observational window may have identified different effects of BFP coverage over time, with important impacts on neonatal mortality as well, which is consistent with a higher number of prenatal visits.

A study pointed out that in 1990 post-neonatal mortality (infant deaths occurred between the 28th to 364th day of life) represented about 44% of the total U5MR, while in 2015, early neonatal mortality (ENMR: infant deaths occurred between birth and the seventh day of life) was the main component of child mortality in Brazil, representing 41% of total deaths [49]. Thus, the findings suggest that there have been changes regarding the age structure of child deaths in recent years.

### Prenatal visits, access to health professionals, and infant mortality

Another point that may explain differences in previous studies regarding the controversy of increased or decreased prenatal visits in the Northeast states and neonatal mortality, is the fact that the number of live births per prenatal visits, as a proxy of the quality of prenatal care provided, is statistically significant for neonatal mortality only. In this sense, there may be a confounding factor involving the results of previous studies, considering that not only the number of prenatal visits, but also the quality of care provided emerges as a major factor in determining neonatal mortality. Our results are in line with a study that stresses the importance of prenatal quality for neonatal mortality [52].

Studies have related neonatal mortality with perinatal causes and, although prenatal care represents a protection factor, mortality is strongly associated with the availability of primary care physicians [24]. In this sense, in our methodological proposal, we considered the overall access to health professionals; physicians, and nurses; as a proxy of the access to comprehensive healthcare. Our findings suggest that in addition to prenatal care, access to health professionals is substantially related to all infant mortality indicators and is better adjusted for IMR and U5MR.

### Fertility rate and infant mortality

The fertility rate was significant for infant mortality and under-five mortality rates. As mentioned in the Method section, this covariate was used as a control variable but also, under the perspective of the capabilities approach, indicates the ability of the household to make reproductive choices. Our results are supported by Barufi et al. [53]. In a quantitative study based on municipal data from 1980 to 2000 in Brazil, the authors indicated that as socioeconomic inequalities such as income and women illiteracy grow, infant mortality also tend to increase and that would be related to adolescent fertility rate that is positively associated with IMR, suggesting that family planning can help to reduce infant mortality. This aspect is very relevant since adolescent mothers are responsible for more than 20% of infants born in Brazil [54] and that there are clusters of adolescent mothers in the North (including Mato Grosso state and Legal Amazon) and the Northeast macro-regions of the country [26].

Our findings are also consistent with studies that have reported a decline in fertility rates accompanied by declines in illiteracy rate and socioeconomic improvements, all related to declines in under-five mortality rates [23, 25, 28, 52]. However, these results should be examined with some caution, since the literature shows that there are conflicting results regarding the association between the birth rate and infant mortality. Reductions in infant mortality and improvements in socioeconomic indicators may hide confounding factors linked to the fertility rate, such as increased female labour market participation, income and education [55].

### Educational attainment and infant mortality

The relation between our dependent variables and educational attainment was statistically significant in all estimations. Educational attainment in our models was applied to evaluate the capacity of households to convert capabilities into functionings.

As part of a criticism formulated by Tengland [20] regarding what the author interprets as a political liberalism conception of health capabilities approach proposed by Sen [18] and Nussbaum [16], job seeking, reproductive health and reproductive choices, as well as education capabilities are part of health as a holistic multi-dimensional phenomenon. Thus, measuring health functioning (infant mortality for instance) is the same as measuring education attainment or employment.

This statement seems to take the capabilities approach to an extreme, however, when exploring the individual’s capabilities, Tengland puts the development of competences as depending on a basic degree of education and special training. In this line, Nussbaum states that as fundamental capabilities, every individual, at least when he or she comes of age, has to be equipped with a decent degree of health and primary and secondary education. In addition, Tengland also stresses that capabilities, in fact, the actualization of capabilities into functionings, is not an excluding or concurrent process. It is possible, and in fact, desirable, for one to actualize multiple capabilities simultaneously, although in some cases some capabilities are not turned into functionings. Thus, it is logical to expect that among households, the capabilities approach suggests that there might be differences concerning their motivation for educational attainment, family planning, and job-seeking as outcomes of the interaction of the dimensions proposed by Ruger, given individual internal characteristics.

This reasoning may also be supported by an apparent contradiction in Nussbaum’s statement regarding her conception of capabilities as plural elements of the quality of life of individuals. Nussbaum stresses that capabilities are qualitatively distinct, such as integrity and bodily health, or education, among other aspects, considered as indivisible and not reducible to a simple metric without distortion. Probably in this statement, Nussbaum was referring to empirical studies aiming to synthesize well-being and happiness in a single scale. In this regard, Anand conducted a study aiming to test the operationalization of variables according to the capabilities approach proposed by Nussbaum based on secondary data from the British Household Panel Survey. The study found evidence suggesting that a wide range of capabilities had a statistically significant association with well-being. The study relied on secondary data sources and subjective well-being concepts according to a scale of life satisfaction from 1 to 7 [56].

Continuing with Nussbaum, the key question to ask when comparing societies is “what is each person capable to do and to be in terms of opportunities?” This reasoning is in line with Tengland’s vision of capabilities as a holistic multi-dimensional phenomenon. Thus, capabilities may be assessed by health and educational attainment at an aggregated level if one intends to assess or to propose public policies aiming to promote a fruitful environment that allows a constant actualization of capabilities. The list of basic capabilities proposed by Nussbaum implies the idea of what the State can do in this sense [16, 20].

### Water supply, sewage services, and infant mortality

Although the lack of a statistically significant association between safe water supply and sewage services and all infant mortality indicators is a controversial result in relation to other studies that found an association between those factors, it is worth noting that most of those studies relied on interpolated data from long periods after the 2000 Census or data covering only part of the municipalities of the country, which may not reflect the evolution of socioeconomic data linked to the sanitary infrastructure.

A study of Guanais based on data of 4853 municipalities of Brazil between 1998 and 2010 found a strong negative association between water supply coverage and infant mortality [28]. That study applied interpolated techniques to obtain data between the National Census from 2001 to 2009, excluding from the analysis a considerable number of municipalities located in rural areas in the North macro-region of the country (*n* = 449) due to the unavailability of socioeconomic data until 2003 [32]. Rasella, in a longitudinal panel data study on the effects of FHS and BFP, used inadequate sanitation coverage as a control variable that encompasses safe water supply and sewage services together [25]. The study was conducted over a three-year period, 2006–2009, with 2853 municipalities of a total 5565, based on interpolated socioeconomic data from the 2000 National Census.

Another possibility for explaining the lack of association between sanitation and water supply and infant mortality indicators may be related to the fact that structural socioeconomic variables change slowly over time in relation to other socioeconomic variables and the changes regarding water supply and sewage services were probably not captured by our model.

We must emphasize that our data related to safe water supply and sewage services coverages are somewhat redundant, as the sum of coverage rates of urban and rural areas exceeds 100%, which suggests that there must exist redundancies in water supply and sewerage systems and/or overreporting errors that must be considered when interpreting our results.

### The health capabilities approach and infant mortality in Brazil

In specific contexts, such as extreme poverty, Sen suggests that one should consider a relatively limited number of central and important functionings and corresponding basic capabilities (such as the ability to be well-nourished and sheltered or escaping from premature death). In other contexts, the number of capabilities and functionings could be much higher and more diversified. One must choose what are the relevant functionings in a specific context and what might be considered negligible [19].

The list of basic capabilities proposed by Nussbaum is far longer than those of our study proposal. Although Tengland reduced this list to a central set of capabilities [20] our methodological proposal restricted those possibilities to the factors interacting with the social and health policies recently implemented in Brazil, to the specificities of the country and data availability.

Tengland’s perspective of health regarding the definition of health capabilities is dynamic in the sense that although some capabilities are impossible for one to convert into functionings, health capabilities must be actualized or turned into functionings. This perspective of health capabilities is more in line with the concept of capability proposed by Nussbaum, for whom capabilities, other than functionings, may be listed as State priorities of actions that may allow individuals to exercise the freedom to choose the life they want to live. On the other hand, for Sen, there is no room for one to define capabilities priorities, and goals. Taking this into account, although Ruger did not make any reference to Nussbaum’s conception of capabilities, the CMHC, and its perspective of health capability as the result of State paternalism and agency seems to be more aligned with a pragmatic perspective of the capabilities approach, having functionings as the “results” to be measured [20].

This study has several strengths. To our knowledge, this is the first study to use the CMHC to study the determinants of infant mortality. The second strength of this study lies in a multi-level data panel with fixed effects nested within-cluster to use aggregate data nested within macro-regions to study the determinants of infant mortality. Third, this is the first time the employment rate is used as an independent variable associated with infant mortality in Brazil. Finally, this study used the longest observational period after the implementation of the BFP in 2003.

### Study limitations

Despite those strengths, this study also has limitations that must be considered when interpreting the results. First, in the application of the CMHC, we were unable to operationalize a variable to control individual characteristics. After conducting a scoping review, we concluded that, in Brazil, regional inequalities can lead to contradictory results such as advanced maternal age as a protective factor for low birth weight. In some regions of the country maternal age conflicts with the level of maternal education. This creates a confounding factor in specific regions of the country. It is a phenomenon known as the “Low Birth Weight Paradox” [57]. On the other hand, we have not found, nor have we been successful in developing a proxy that could be used as a control for the internal dimension in the CMHC. Second, the CMHC was designed to operationalize variables at the individual level and our approach was designed based on aggregated data at the state level. As there is no data available on the employment rate at the municipal level, we use aggregated data at the state level, which can generate a limitation. Although our results are consistent with findings in other studies, the risk of ecological fallacy cannot be ignored, especially with regard to the effects of the variables analyzed at the household level, such as the birth rate and family planning. Third, as we have mentioned in the “Independent Variables” section we applied interpolation techniques for specific periods of our independent variables. Although those are minor interpolations and estimations, they must be taken into account when interpreting our findings. Fourth, in some states, the total coverage of safe water and sewage services exceeds 100%, suggesting the existence of overreporting or more than one contract per household, which should be considered when interpreting the results. Fifth, although the quality of infant mortality information has recently improved in the North and Northeast regions, underreporting still occurs, mainly regarding rural areas and municipalities with small populations, and must be taken into account when interpreting the results of the present study as a possibility source of bias. Finally, there are some limitations regarding the external validity of our study due to the specificities of Brazil. Although the World Bank classifies Brazil as an upper-middle-income country [58], it ranks 73rd in terms of per capita income. The country has a population of about 212 million living in the world’s fifth-biggest territory and is the only Portuguese-speaking country in the Americas. Although in terms of absolute GDP value, Brazil ranked eighth in the world in 2018, economic inequalities in the country have reached extreme levels and are one of the worst in the world.

## Conclusions

The results of this study showed that the CMHC is a useful tool for the analysis of the effects of social determinants of health in an upper-middle-income country with distinct subregional characteristics, under the effects of an inclusive institutional, social and health policies framework.

Our results were only made possible by using the multilevel panel data model with fixed effects nested within-cluster. The method allowed the use of the variables provided by the conceptual framework based on aggregated data that could hardly be used by other methodologies without leading to incorrect estimations. The estimations could isolate the effects of the variables under study from factors not observed, which are subject to estimation errors due to different degrees of error homogeneity within and between clusters.

Our models covered a longer observational window that allowed us to infer more about specific factors related to infant mortality, such as the relation between the employment rate and different indicators of infant mortality and the BFP and the neonatal mortality rate or the threshold of household income according to minimum wage bracket which acts as a protective factor for infant mortality.

Furthermore, the use of the methodology of clustered observations at different levels of fixed effects is a low cost-benefit solution, considering that it relies on a low volume of data when compared to conventional panel data studies.

## Supplementary Information

**Additional file 1: Appendix 1.** Correlation Matrix. **Appendix 2.** Estimation with fixed effect clustering by “macro-regions” and “year", absorbing “year”

## Data Availability

The datasets used and/or analyzed during the current study available from the corresponding author on reasonable request.

## Footnotes

1 Information available at the National Employment Survey (IBGE-*Pesquisa Mensal de Emprego*): ftp://ftp.ibge.gov.br/Trabalho_e_Rendimento/Pesquisa_Mensal_de_Emprego/Metodologia_da_Pesquisa/srmpme_2ed.pdf.

2 Information available at http://www.ibge.gov.br.

3 Information available at http://www2.datasus.gov.br/DATASUS/index.php?area=02.
