# Supplementary material for: Health capabilities and the determinants of infant mortality in Brazil, 2004–2015: an innovative methodological framework
Source: BMC Public Health. 2021 Apr 30;21:831. doi: 10.1186/s12889-021-10903-9 (PMC8086285; doi:10.1186/s12889-021-10903-9)
Supplement: Supplementary file 1 — Additional file 1: Appendix 1. Correlation Matrix. Appendix 2. Estimation with fixed effect clustering by “macro-regions” and “year", absorbing “year” [file 12889_2021_10903_MOESM1_ESM.docx]

**Appendices**

# Health Capabilities and the Determinants of Infant Mortality in Brazil, 2004-2015: an innovative methodological framework

Alexandre Bugelli^1^* - Roxane Borgès Da Silva^2^ - Ladislau Dowbor^3^ - Claude Sicotte^4^

***Appendix 1 Correlation Matrix***


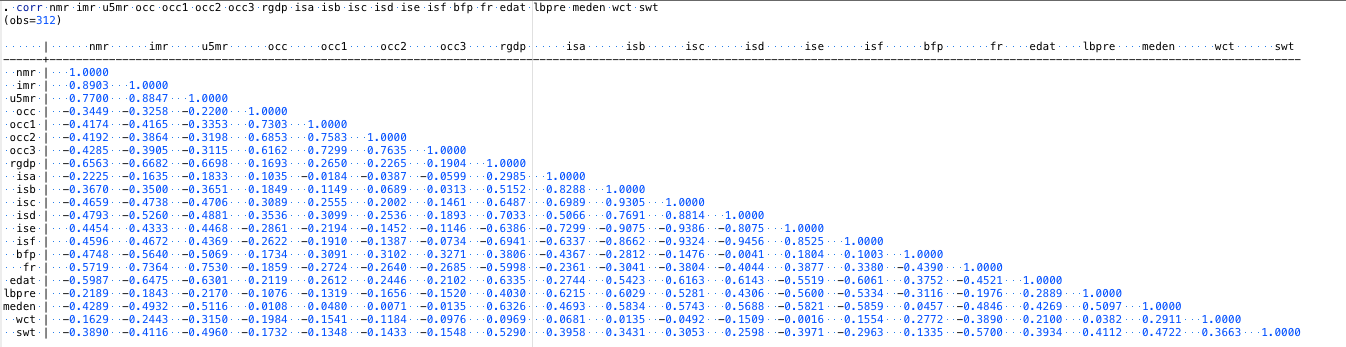


Appendix 2 Estimation with fixed effect clustering by

"macro-regions" and "year", absorbing "year"

**
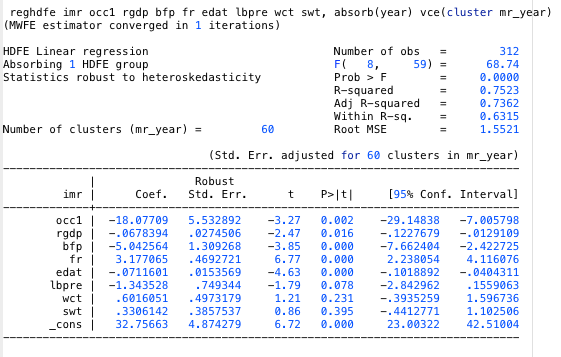
**
